# A Characterization of Brain-Computer Interface Performance Trade-Offs Using Support Vector Machines and Deep Neural Networks to Decode Movement Intent

**DOI:** 10.3389/fnins.2018.00763

**Published:** 2018-10-24

**Authors:** Nicholas D. Skomrock, Michael A. Schwemmer, Jordyn E. Ting, Hemang R. Trivedi, Gaurav Sharma, Marcia A. Bockbrader, David A. Friedenberg

**Affiliations:** ^1^Advanced Analytics and Health Research, Battelle Memorial Institute, Columbus, OH, United States; ^2^Medical Devices and Neuromodulation, Battelle Memorial Institute, Columbus, OH, United States; ^3^Neurological Institute, The Ohio State University, Columbus, OH, United States; ^4^Department of Physical Medicine and Rehabilitation, The Ohio State University, Columbus, OH, United States

**Keywords:** brain-computer interface, decoding, machine learning, deep learning, support vector machines, response time

## Abstract

Laboratory demonstrations of brain-computer interface (BCI) systems show promise for reducing disability associated with paralysis by directly linking neural activity to the control of assistive devices. Surveys of potential users have revealed several key BCI performance criteria for clinical translation of such a system. Of these criteria, high accuracy, short response latencies, and multi-functionality are three key characteristics directly impacted by the neural decoding component of the BCI system, the algorithm that translates neural activity into control signals. Building a decoder that simultaneously addresses these three criteria is complicated because optimizing for one criterion may lead to undesirable changes in the other criteria. Unfortunately, there has been little work to date to quantify how decoder design simultaneously affects these performance characteristics. Here, we systematically explore the trade-off between accuracy, response latency, and multi-functionality for discrete movement classification using two different decoding strategies–a support vector machine (SVM) classifier which represents the current state-of-the-art for discrete movement classification in laboratory demonstrations and a proposed deep neural network (DNN) framework. We utilized historical intracortical recordings from a human tetraplegic study participant, who imagined performing several different hand and finger movements. For both decoders, we found that response time increases (i.e., slower reaction) and accuracy decreases as the number of functions increases. However, we also found that both the increase of response times and the decline in accuracy with additional functions is less for the DNN than the SVM. We also show that data preprocessing steps can affect the performance characteristics of the two decoders in drastically different ways. Finally, we evaluated the performance of our tetraplegic participant using the DNN decoder in real-time to control functional electrical stimulation (FES) of his paralyzed forearm. We compared his performance to that of able-bodied participants performing the same task, establishing a quantitative target for ideal BCI-FES performance on this task. Cumulatively, these results help quantify BCI decoder performance characteristics relevant to potential users and the complex interactions between them.

## Introduction

Intracortical brain-computer interface (BCI) systems that link neural activity to the control of assistive devices have the potential to reduce disability associated with paralysis (Lebedev, 2014; Chaudhary et al., 2016). Recent years have seen numerous demonstrations of BCI control, including computer cursors, robotic arms, communication devices, and even the patients' own paralyzed limbs (Simeral et al., 2011; Hochberg et al., 2012; Collinger et al., 2013b; Gilja et al., 2015; Jarosiewicz et al., 2015; Bouton et al., 2016; Ajiboye et al., 2017). In anticipation of BCI systems transitioning from laboratory demonstration to clinical usage, it is important to consider the priorities of the end user to ensure widespread adoption. Surveys of potential users have revealed that high accuracy, fast response times, and multi-functionality are among the most desired features for a BCI system (Huggins et al., 2011, 2015; Collinger et al., 2013a; Kageyama et al., 2014). Designing BCI systems to meet these priorities may facilitate the adoption of these systems for every day, clinical usage.

Accuracy, response latency, and the number of functions provided by a BCI system are all directly affected by the neural decoding component of the system (Kao et al., 2014; Lebedev, 2014). The decoding algorithm is responsible for translating the user's neural activity into an intended action that is selected from a set of possible functions. As a result, the decoding algorithm is tied not only to the number of functions/actions that can be decoded, but also to how accurately and how fast they can be decoded. Additionally, the response time–the time between the user intending to act and the BCI identifying the user's intention–is crucial for BCI user's sense of agency (the feeling of being in control of the BCI action; Evans et al., 2015; Moore, 2016; Sitaram et al., 2017).

Designing a decoder that meets BCI-user expectations for accuracy, response time, and number of functions first requires establishing minimal acceptable criteria for each feature. By surveying potential BCI users with spinal cord injuries, Huggins et al. (2015) found that the majority of respondents would be satisfied with an accuracy of 90% or above. Although this can be treated as a minimal acceptable criterion, one must keep in mind that accuracy can have different meanings depending upon the BCI-enabled task (Thomas et al., 2013; Thompson et al., 2014). For example, BCI systems for continuous cursor control can be evaluated using several different metrics, such as the correlation between the predicted and actual cursor movement (R^2^) (Simeral et al., 2011; Nuyujukian et al., 2014; Sussillo et al., 2016). On the other hand, BCIs used for discrete control signals, e.g., “on” “off,” or “left” “right,” are typically evaluated using standard classification accuracy–the percent of time bins where the decoder correctly classifies the discrete function (e.g., Bouton et al., 2016). As such, the minimal acceptance criterion for BCI accuracy may be task-dependent. Huggins et al. also found that their respondents desired a target response time of at least 20–24 characters per minute (2.5–3 s per response) for BCI communication systems. Currently, no specific latency criteria exist for BCI devices aimed at restoring hand function. However, studies of cursor control, via imagined hand movements with EEG-based BCI systems in able-bodied participants, suggest that users experience a decline in sense of agency with delays as short as 750 ms (Evans et al., 2015). Lastly, while potential users prioritized the number of available BCI functions, the surveys did not suggest a target number that users would find acceptable. In the absence of specific data, we aim to maximize the number of functions while still meeting performance expectations for accuracy and response time.

Simultaneously addressing these three user-desired performance priorities for a BCI decoder is complicated by the fact that they represent competing demands. For example, in BCI virtual keyboard communication devices, increased accuracy of key selection can come at the price of slower response times (Santhanam et al., 2006). Similarly, for BCI cursor control, increased accuracy is associated with decreased speed (Willett et al., 2017). Additionally, BCI systems with discrete control signals often suffer from decreased accuracy when the number of overall functions are increased (Thomas et al., 2013). To address these issues, we systematically explore the trade-off between these three performance criteria in a BCI system that decodes discrete hand movement from intracortical neural recordings. To do so, we use two different decoding strategies–a support vector machine (SVM) classifier which represents a commonly used method for BCI decoding (e.g., Lotte et al., 2007; Siuly and Li, 2012; Bouton et al., 2016; Friedenberg et al., 2016; Sharma et al., 2016; Glaser et al., 2017; Colachis et al., 2018) and a deep neural network (DNN) framework (Schwemmer et al., 2018). Building on the recent work by Schwemmer et al. (2018), we evaluate the decoders using historical intracortical recordings from our participant with tetraplegia where he imagines performing several hand and finger movements. We find that response time increases and accuracy decreases as the number of functions increases for both decoders. However, we show that this slowing of response times and decline in accuracy with added hand functions is significantly less for the DNN than the SVM. Interestingly, we find that data preprocessing steps affect the response times of the two decoders in different ways. We also show that the DNN decoder can be used in real-time to control functional electrical stimulation (FES) of the participants' paralyzed forearm, allowing him to perform six different hand/finger movements. Finally, to establish quantitative benchmark that can be used to evaluate the performance of the BCI-FES system against an ideal target we collected data from three able-bodied individuals who performed the same six-movement task. These results help characterize BCI decoder performance features relevant to potential users and the complex interactions between them.

## Materials and methods

### Study design–clinical trial participant

The study (ClinicalTrials.gov NCT01997125) was approved by the U.S. Food and Drug Administration (Investigational Device Exemption G130055) and the Ohio State University Wexner Medical Center Institutional Review Board (IRB Protocol 2013H016, OSUWMC, Columbus, Ohio) and conformed to institutional requirements for the conduct of human subjects. All experiments were performed in accordance with the relevant guidelines and regulations set by OSUWMC. The participant referenced in this work provided permission for photographs and videos and completed an informed consent process prior to commencement of the study.

**Figure 1:**
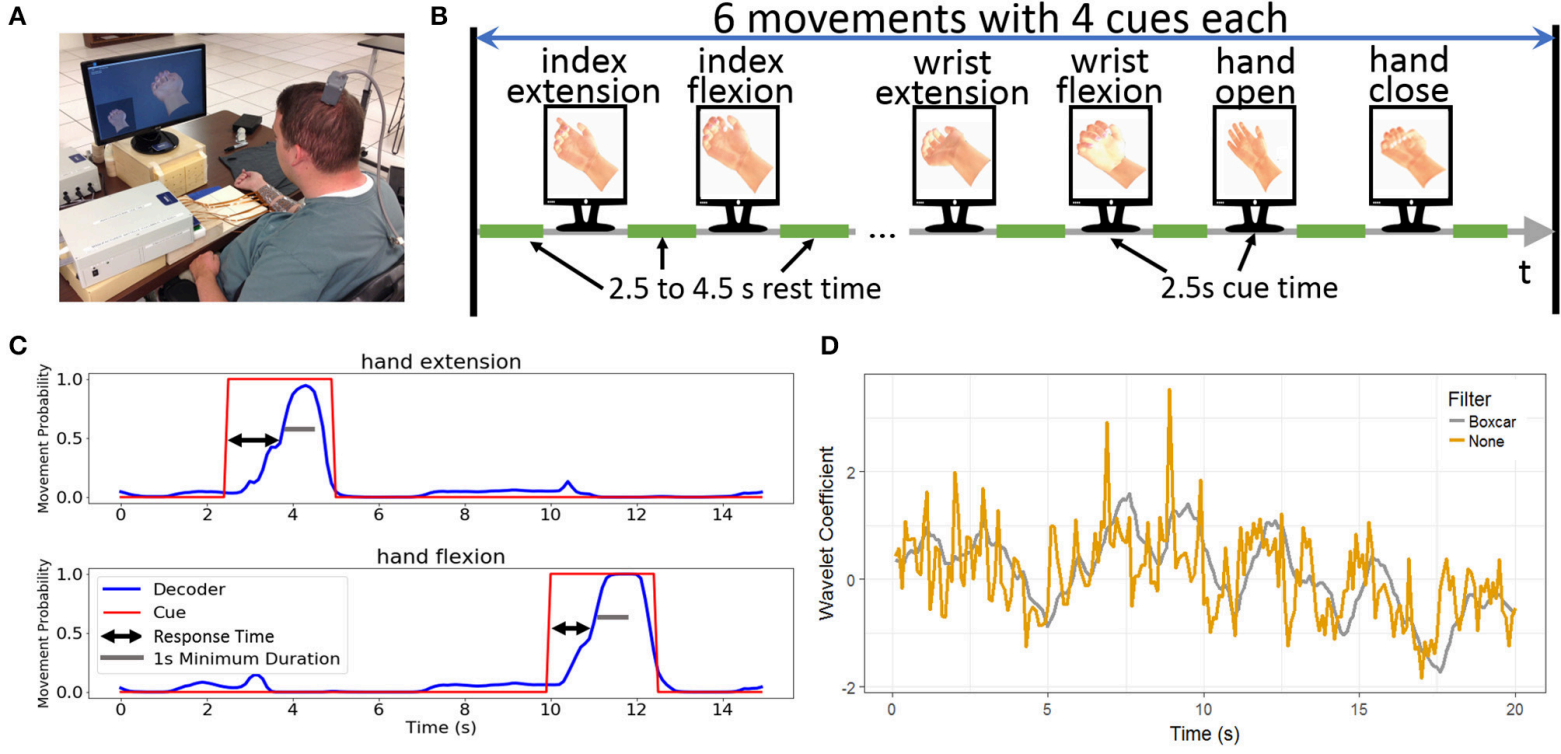
Experimental design. **(A)** The participant performing the six-movement task using the BCI-FES system. The cued movement is displayed and the participant is asked to imagine replicating the cued movement. **(B)** Design of the six-movement task where each movement is repeated for a total of four replicates in random order. Each cue remains for 2.5 s with variable rest time of 2.5–4.5 s between the cues. **(C)** Sample decoder output demonstrating the response time. Response time is the difference between the start of the cue to when the decoder output initiates the correct movement. To be successful, the movement must be sustained for a minimum of 1 s. **(D)** Impact of the boxcar filter. The yellow line shows the wavelet coefficient across time for a single channel and wavelet scale, whereas the gray line shows the same data with the boxcar filter applied. While the two lines track the same general trends, there is substantially more variation without the filter.

The study participant has a C5 AIS category A traumatic spinal cord injury acquired 4 years prior to the initiation of the study. He is currently 26 years old. On April 22, 2014, A Utah microelectrode array (Blackrock Microsystems, Inc., Salt Lake, Utah) was implanted in the hand area of his left primary motor cortex as previously described (Bouton et al., 2016). During the experiments reported in this manuscript, a computer monitor displayed two animated hands to the participant (Figure 1A). For the imagined movement tasks, the cues to think about specific movements were given by a small hand in the lower left corner of the screen. During rest periods, the hand remained in a neutral position. During experiments where the participant had volitional control of functional electrical stimulation and was given visual feedback, the larger hand centered on the screen provided real-time feedback from the BCI system. Otherwise, the feedback hand remained in a neutral resting position.

### Datasets for offline analyses

For offline analyses, data was collected from two separate tasks where the participant imagined making hand/finger movements without controlling FES or receiving visual feedback on performance. These imagined tasks allow for the independent evaluation of algorithms without the confounding effect of the participant adapting to the decoder that is generating the feedback. The two-movement task cued the participant to imagine hand open or hand close for 2.5 s separated by 6.5 s of rest (the rest cue showed the virtual hand in a neutral position). Each movement was replicated five times and the presentation of the cues was randomly shuffled to prevent the participant from anticipating the next cue. We called this entire 90 s (25 s of cues and 65 s of rest) sequence a block. The four-movement task cued the participant to imagine performing index extension, index flexion, wrist extension, and wrist flexion. Each movement cue lasted for 2.5 s and movement cues were separated by 4 s of rest. Each movement was repeated four times and, as with the two-movement task, the movement cues were randomly shuffled. Each block of the four-movement task lasts 104 s (40 s of cues and 64 s of rest). Figure 1B shows an illustration of the structure for a typical block.

Two blocks of both the four- and two-movement experiments per session were collected during fifty experimental sessions ranging from June 1, 2016 to November 28, 2017. The first forty sessions were treated as training data and the remaining ten sessions were considered the testing data.

### Definitions of key metrics

The result of a cued movement was considered to be successful if the correct movement was predicted and sustained for a minimum of 1 s within the 2.5 s cue time window (Figure 1C). The success rate for an experimental block was defined as the percentage of cues in the block that were successes. The failure rate was the percentage of cues that are not successes. Ensuring that the correct response was sustained allowed us to filter out functionally irrelevant situations where the decoder correctly predicts the cue for a brief window and then switches to predicting a different, erroneous movement. To further quantify decoder performance, we also calculated the decoder accuracy, defined as the proportion of 100 ms time bins where the decoder correctly predicted the cued hand movement.

To demonstrate the different accuracy metrics consider the 90 s two movement task which has ten movement cues. The success rate is the percentage of the 10 cues where the correct movement was predicted continuously for at least 1 s. In contrast, the accuracy is the percentage of the 900 time bins (90 s × 10 time bin/s) where the decoder prediction matched the cue. The success rate is meant to approximate an observer scoring each movement cue as a success or failure, whereas the accuracy is the standard machine learning classification accuracy.

Response time was defined as the time between the start of the cue to the initiation of a successful movement (where success was defined as above). In offline analyses, the initiation of the successful movement was determined by the first 100 ms time bin where the decoder predicted the correct movement and sustained the correct prediction for at least 1 s (Figure 1C) In the real-time demonstrations, where hand movements were evoked, the initiation of the correct hand movement was calculated from the first frame of video where the hand began moving toward the cued position for a successful movement. Note that for both the response time and success rate, the first cued movement was ignored because the DNN starts predicting during the middle of cue due to the time lagged input.

### Data acquisition and preprocessing

Data was collected at 30 kHz from the 96 channel Utah microelectrode array with 1.5 mm electrodes using the Neuroport™ neural data acquisition (Blackrock Microsystems). A 0.3 Hz first order high-pass and a 7.5 kHz third order low-pass Butterworth analog hardware filters were applied to the data. The data were then transferred to a PC running Matlab R2014b and Python 2.7 for further processing.

The raw data was reduced using wavelet decomposition with the “db4” mother wavelet and 11 wavelet scales in Matlab R2014b. Features for decoding were created by extracting the wavelet coefficients for scales 3, 4, and 5, spanning frequency range 234–1,875 Hz. Every 100 ms, the coefficients were averaged over time, providing 96^*^3 = 288 features per bin. Next, these averaged wavelet coefficients for each channel were individually standardized over a single block of data. During the training period, each block of data was standardized to itself, while during the testing period, the mean and standard deviation of the first block was used to standardize both the first and second blocks. Once the 288 features were standardized, the 3 averaged and standardized coefficients for each channel were then averaged together to create a single feature, called Mean Wavelet Power (MWP), resulting in 96 features, one per channel, for each 100 ms time bin. Previous work from our group has demonstrated the success of using MWP in decoding movement intent (Sharma et al., 2015, 2016; Bouton et al., 2016; Friedenberg et al., 2017; Colachis et al., 2018).

We performed an additional preprocessing step where the MWP features for each channel were smoothed by replacing the current time point with the average of the most recent 10 time points (i.e., a 1 s boxcar filter, see Figure 1D) on a subset of analyses to determine the effect of this preprocessing step on the two different decoders.

### Neural decoding and offline analyses

We evaluated two different decoding algorithms with respect to their impact on both accuracy and response time. The first decoding algorithm is a support vector machine (SVM) classifier that we have used previously (Bouton et al., 2016; Friedenberg and Schwemmer, 2016; Sharma et al., 2016). The SVM is trained *de novo* each day during the testing period using only the first block of data and evaluated on the second block of data from the same day (Figure 1D). That is, the SVM is only ever trained on a single block of data, but it is retrained every single session. The SVM uses nonlinear Gaussian radial basis functions kernels with a γ parameter value of 0.005 and uses the MWP features at the current time point only in order to predict the intended movement. The SVM was trained using the sci-kit learn toolbox (Pedregosa et al., 2011) in Python 2.7 using the default parameter values except for the value of the γ which we based on our previous experience using the SVM. The SVM retrained daily is used as the benchmark comparison since it reflects the standard of practice for this clinical study and daily retraining is the standard mode of operation for most intracortical BCI systems.

The second decoding algorithm is a deep neural network (DNN) decoder constructed and trained with Python 2.7 using the package Keras (Chollet, 2015) with TensorFlow™ (Abadi et al., 2016) as the backend. The architecture of the network is similar to one used in our previous work (Schwemmer et al., 2018). The network takes as input a 96 × 9-dimensional array corresponding to 900 ms of MWP data. That is, the model uses a sliding window of 900 ms to predict the imagined movement for the current time point. The first layer in the network is a long short-term memory (LSTM) layer (Hochreiter and Schmidhuber, 1997) containing 80 hidden units. The LSTM is a variant of a recurrent neural network that is capable of learning long-term dependencies and has been widely used in the processing of temporal (or sequential) data (LeCun et al., 2015; Goodfellow et al., 2016). The LSTM layer outputs an 80 × 9-dimensional array that is passed to a one-dimensional convolutional layer that contains twenty-five 80 × 9-dimensional filters. The convolution is performed in the time domain only. The output of this layer is then flattened to a 225-dimensional vector which is then passed to a fully connected (dense) layer with 50 units using the rectified linear unit activation function. The output from this layer is passed to a final dense layer containing units equaling the total number of movements plus rest. The units in this final layer use the softmax activation function scaling the outputs to correspond to probabilities.

The 80 blocks from the first 40 sessions of the two- and four-movement tasks were used to train the DNN model. The DNN was trained using random batches of size 200 using the optimizer RMSprop (Ruder, 2016) and the categorical cross-entropy loss function. All network parameters were randomly initialized at the start of the training using the Keras defaults. During each training epoch, each layer in the model underwent a random 50% dropout of the connection weights in order to prevent overfitting to the training data (Srivastava et al., 2014). The training lasted for 80 epochs and was completed using an NVIDIA Quadro K5000 GPU on a Linux system running Centos 7.3. The 80 epochs of training was based upon the model reaching a stable loss and accuracy value for the validation data during training. Subsequently, the number of training epochs was treated as a fixed hyperparameter (at 80) to prevent overfitting to the training dataset. The DNN's performance was then evaluated on the second blocks of each of the last 10 sessions.

### Simulating datasets with different numbers of movements

To quantify the trade-off between accuracy, response-time, and the number of movements, the two- and four-movement datasets were concatenated in each session, creating an aggregate dataset with six separate movements. To create datasets that contained fewer movements, we excised data corresponding to different cues and created a series of synthetic datasets that covered the range of all possible combinations of the six hand movements (ranging from one to six movements). For example, there were six different one-movement datasets, one for each of the six movements, and one six-movement dataset. For each of the two through five-movement datasets, we created a synthetic dataset for every permutation of the different movements. In these synthetic datasets, data associated with cues for excluded movements were removed but all rest cues were retained.

### Real-time demonstration of the six-movement task

In this experiment, the DNN model decoder controlled functional electrical stimulation (FES) of the participants' paralyzed forearm. The FES system consists of a multi-channel stimulator and a flexible sleeve consisting of up to 150 electrodes that is wrapped around the participant's arm. Offline, electrode stimulation patterns corresponding to the six movements in this task were calibrated using knowledge of forearm physiology and trial and error. A six-movement task was performed using the same movements in the previous sections but with random rest intervals varying from 2 to 5 s and a cue time of 2.5 s. Each cue was repeated four times for a total of 24 movements per block. A DNN model was first trained offline using the concatenated imagined six-movement dataset and was updated for 15 epochs using cued data from a single block. This model was then used for five blocks of the six-movement task within a single session on January 15, 2017. The same model was then updated using the five blocks of collected data and trained for 10 epochs. This updated model was then used in a subsequent session to make predictions on the participants intended hand movements to direct the FES system.

On January 22, 2018 the participant performed three blocks of the six-movement task to assess his response times when using the BCI-FES system. The time from the start of the cue to the initiation of the hand movement was measured from using video data with a timer displayed on the monitor (Movie S1). Response time measurements were recorded as the time from cue presentation to initiation of the correct hand movement based upon the stopwatch in the video recording. Additional response times were computed for the tetraplegic participant using the decoder outputs as previously described.

### Able-bodied six-movement task

To establish a baseline for movement response times that could be used to quantitatively compare the response time of the BCI-FES system, three able-bodied participants also performed the six-movement task under an IRB-exempt protocol approved by the Battelle Memorial Institute (Columbus, Ohio). The three participants ranged in ages from 20 to 25 and volunteered to participate in the demonstration. The able-bodied participants were asked to perform the same six-movement task and were cued using the same animated hand and timing profiles described above. They were instructed to mimic the hand movements in a “natural and comfortable” manner. Each participant performed 3 blocks of the six-movement task. Videos were recorded and the time from the start of the cue to the initiation of the hand movement was measured in the same manner as described for the clinical trial participant. The results were aggregated across the participants and blocks to obtain average able-bodied response times for a given hand movement.

### Statistical analyses

Analyses to compare the success rate or accuracy between paired observations were done with a Wilcoxon signed rank test. Comparisons across multiple groups were completed using generalized linear or ANOVA models. Analyses comparing regression slopes were conducted by the F-test for significance of slopes. All model assumptions were explored graphically, and data is presented to validate these assumptions.

## Results

### Comparison of decoding and data preprocessing methods

**Figure 2:**
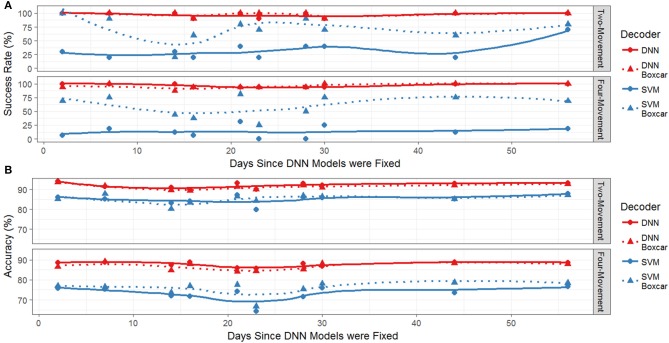
**(A)** Success rate for both the two-movement and four-movement offline tasks during the testing period. The redlines represent the performance of the DNN and the blue line represents the SVM. Solid lines and circles use the boxcar filter and dashed lines with triangles are without the filter. **(B)** Accuracy as a function of days since last training session. The redlines are the performance of the DNN and the blue line is the performance of the SVM. Solid lines and circles are decoders using the boxcar filter and dashed lines with triangles are without the boxcar filter.

We first explored the differences in success rates and response times for the two decoding methods on the two- and four-movement tasks offline. Figure 2A plots the success rate (% of cued movements where the model correctly predicts and sustains the correct movement for at least 1 s) of the decoders (SVM and DNN) during the testing period as a function of the number of days since the end of the initial training period for the DNN. Recall that the DNN is trained on 40 sessions of historical data and then held fixed during the testing period while the SVM is retrained each day. Also shown is the success rate of the models when an additional preprocessing step (replacing the MWP at the current time point with the average of the most recent 10-time points, i.e., a 1 s sliding boxcar filter) is applied to the data prior to being input to the decoders. We have included this additional comparison (boxcar on vs. boxcar off) since we have previously found that including the boxcar filter is key to the SVM's decoding performance. This is illustrated in Figure 2A, which shows that the success rate of the SVM without the boxcar (dashed blue lines) was significantly less than the SVM with the boxcar (solid blue lines). The average success rate for the SVM on the four-movement (two-movement) task with the boxcar was 60.0 ± 19.3 (71.1 ± 23.0, mean ± s.d.) while the success rate without the boxcar was 12.7 ± 11.5 (28.9 ± 17.5). In contrast, the success rate of the DNN appeared to be unaffected by the inclusion of the boxcar filter preprocessing step (compare solid and dashed red lines). A two-way ANOVA of the success rate fit against the model type and the presence of the boxcar filter found that there were significant differences between both model type and use of the boxcar filter (*p* < 0.001), except there was not a significant difference between the boxcar on and off DNN models. This finding was consistent for both the two- and four-movement tasks. The DNN's success rate without the boxcar (with the boxcar) was 97.3 ± 3.4 (96.0 ± 4.7) on the four-movement task and 96.7 ± 5.4 (97.8 ± 4.7) on the two-movement task. The classification accuracies (% of correctly predicted time bins) shown in Figure 2B showed the same pattern. The DNN (SVM with boxcar) accuracy was 87.8 ± 1.3 (75.9 ± 3.6) on the four-movement task and 91.9 ± 1.5 (85.2 ± 2.2) on the two-movement task.

**Figure 3:**
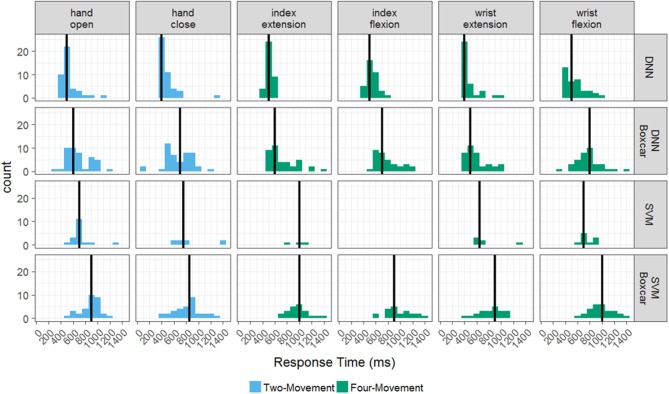
Histograms of response times for the offline tasks. The distribution response times for each successful hand movement across the testing period, broken out by decoder-filter combination and task. The two-movement task is in blue and the four-movement task is in green. Mean response times for a decoder-filter combination and hand movement are represented by solid black lines.

Response times also significantly varied for each of the four models as shown in Figure 3. Recall that the response time was calculated as the time from cue onset until the model predicts the correct cue and sustains it for at least 1 s. Figure 3 plots histograms of the model responses times broken out by the individual movements from the two- and four-movement tasks. Even though the response times are aggregated by individual movement, note that the movements were performed in different tasks (as indicated by the coloring of the histograms). The black vertical line indicates the mean of each distribution. Although we found the success rate for the DNN was not significantly different whether or not the boxcar filter was used, there is a clear increase in response time when using the boxcar filter for both tasks (*p* < 0.001). The mean response time for the DNN across all movements on the four-movement (two-movement) task without the boxcar was 524 ± 128 ms (506 ± 150 ms), whereas the mean response time with the boxcar was 719 ± 218 ms (683 ± 211 ms). This makes intuitive sense as the boxcar filter averages past information in with the most current information (see Figure 1D), which smooths the data but can slow the model response times. As we have shown, this tradeoff is necessary for the SVM to accurately decode the imagined movements but is not required for the DNN.

The mean response time of the SVM with the boxcar filter on the four-movement (two-movement) task was 946 ± 205 ms (855 ± 196), which was significantly slower than both DNN models. Comparisons to the SVM without the boxcar filter were not conducted due to the poor success rate of the models (12.6 and 28.9% for the four-movement and two-movement tasks). These results show that the DNN model does not require the additional preprocessing step of the boxcar filter and has both higher accuracy and faster response times than the SVM.

### Characterizing performance trade-offs via simulation

In the previous section, we compared the response times, success rates, and accuracies of the two decoding algorithms on both the two- and four-movement tasks offline. Although these results provide insight into how the number of decoder functions (two vs. four) affects performance, it does not provide a full characterization of the trade-offs that may exist between the three metrics. Thus, we conducted a simulation experiment which allowed us to use historical data from the two- and four-movement tasks to more fully quantify the performance characteristics as a function of the number of movements. As detailed in methods, the data sets from both tasks were concatenated for each experimental session yielding a synthetic six-movement task. We then excised data by movement from each set and created additional synthetic datasets ranging from each movement individually to all six movements. For the datasets that contained <6 movements, all possible permutations of movement combinations were extracted. For example, the one movement dataset consisted of each individual movement where data corresponding to all other movements were removed (i.e., one block of the six-movement task yielded 6 separate one-movement blocks). Next, for the two movement datasets, a dataset was created for each possible pair of movements. This was repeated for three, four five, and six movements leading to synthetic datasets for all possible permutations. Decoders were trained and tested for each movement combination and then aggregated according to the total number of movements. We chose to compare the SVM decoder with the boxcar filter and the DNN decoder without the boxcar filter as they were best performing combination for each algorithm from the previous section.

**Figure 4:**
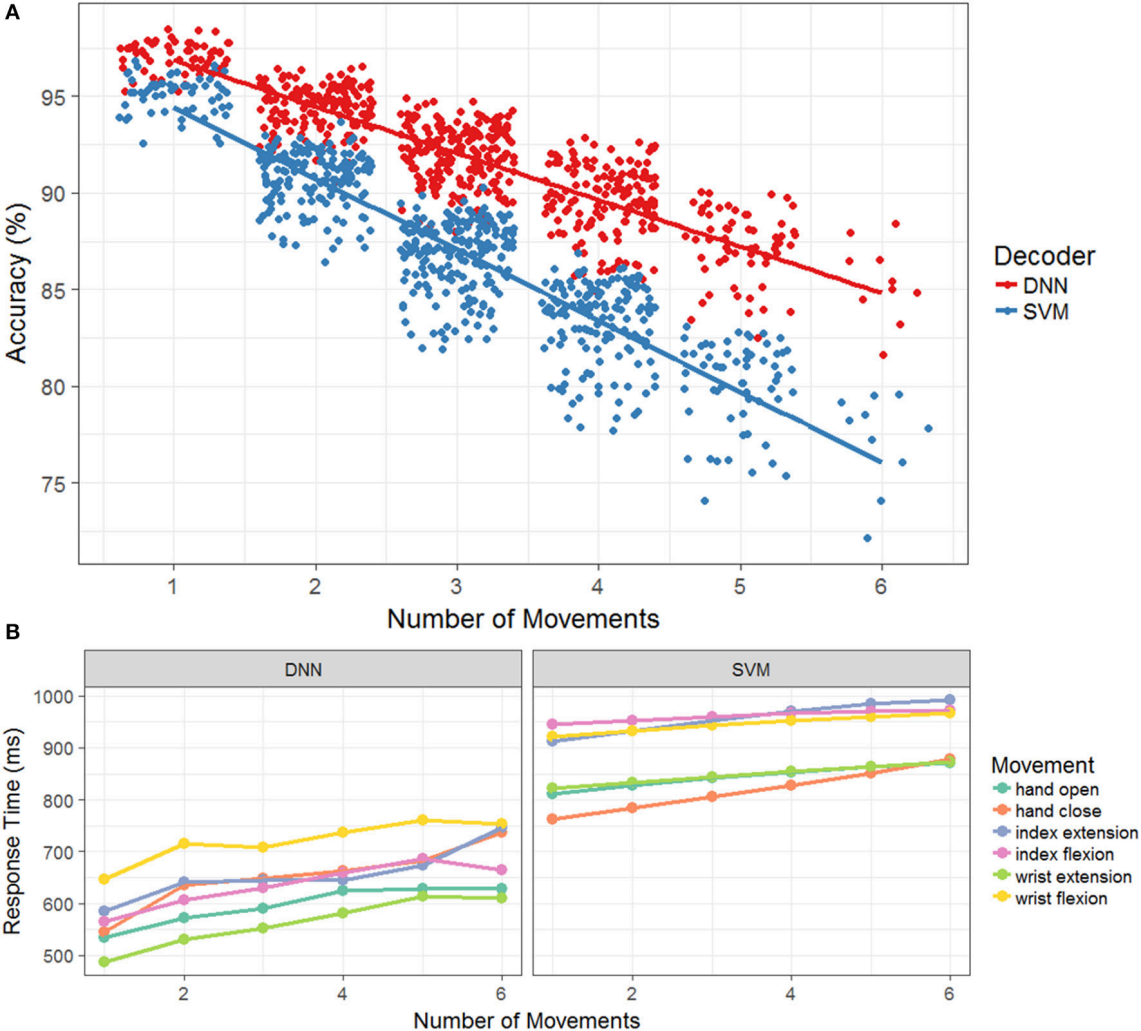
**(A)** Accuracy as a function of the number of movements during the offline simulation. The accuracy across each of the testing days in the simulation, with the DNN is in red and the SVM is in blue. A jitter is applied to easy visualization and a linear regression model is fit to the original data. **(B)** Response time as a function of the number of movements during the offline simulation. The mean response time of successful movements for the DNN and SVM color coded by the different hand movements. As the number of movements increase, so does the mean response time for both model types.

Figure 4A plots the model accuracies for each test session as a function of the number of total movements. Note that even though there are only 10 test sessions, the number of data points shown in the figure can vary for the different numbers of movements owing to the fact that there are $\left(\begin{array}{l} 6\\ k \end{array}\right)$ combinations for each total number of movements *k*. The data points in the figure are also randomly jittered in the x-coordinate for easier visualization. Overall, model accuracy during the testing set declined as the number of movements increased for both decoders. However, the rate of the decline was significantly greater (*p* < 0.001) for the SVM (slope = −3.7, *p* < 0.001) than the DNN (slope = −2.4, *p* < 0.001), indicating a relatively greater cost to accuracy for additional movements with the SVM compared to the DNN. Next, we explored how the individual movement response times changed as the number of total movements increased. Figure 4B plots the average response time (averaged across testing sessions and movement combinations) as a function of the number of total movements, broken out by the specific movement and the decoding model. As expected, response time increased with the number of movements (DNN: slope = 23.8, *p* < 0.001; SVM: slope = 11.82, *p* < 0.001). The slope of the SVM does appear to be smaller than the slope of the DNN, but the difference was only marginally significant (*p* = 0.046) based upon a likelihood ratio test comparing models with and without separate slopes for decoder. Although the timing varied by the movements themselves, the rates of change were not significantly different across the movements. The DNN consistently had faster average response times than the SVM across all movements. Additionally, the average response times across testing sessions, movement combinations, and individual movements ranged from 566 ± 130 to 694 ± 55 ms for the DNN and 862 ± 130 to 914 ± 63 ms for the SVM. For this task, increasing the number of total movements clearly leads to increased response times and decreased accuracy for both the DNN and SVM.

**Figure 5:**
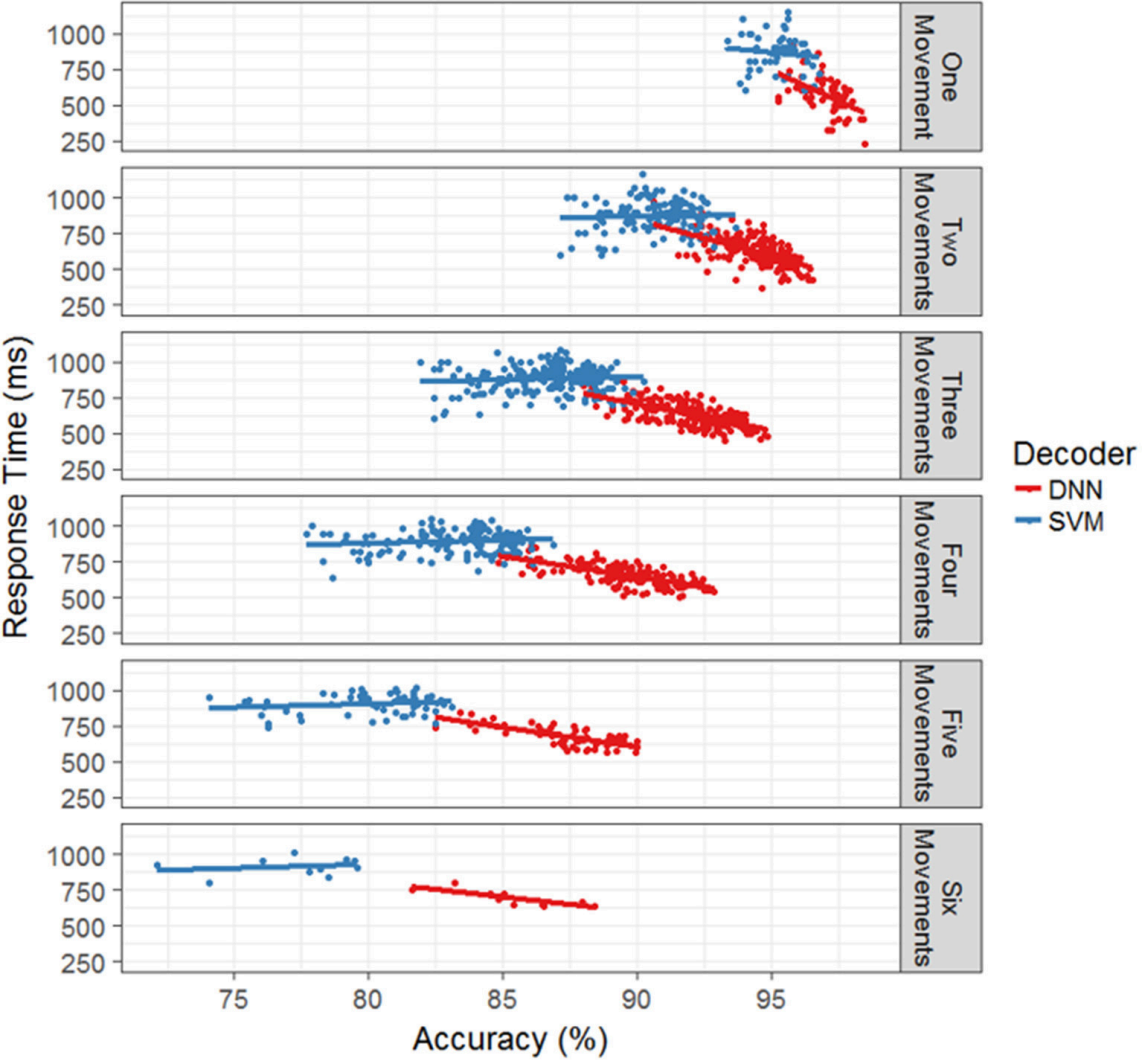
Response time as a function of accuracy for the offline simulation. For each number of hand movements in the simulation, the mean response time is plotted against the accuracy for each separate test days. The SVM with the boxcar is in blue and the DNN is in red. Linear regression models were fit to each set of points to provide interpretation of the trends. For the SVM, all of the models appear to be flat, allowing for large variation in accuracy while maintaining similar response times. For the DNN, the trend shows a decrease in response time with an increase in accuracy.

Lastly, we investigated whether the models displayed a speed-accuracy trade-off in terms of their response times. Figure 5 plots the average model response time as a function of accuracy for each test session broken out by model type and the number of total movements. The SVM does not appear to have a significant interaction between accuracy and response time while the DNN displays a negative interaction. That is, the faster responses correlate with more accurate responses, which makes intuitive sense since the accuracy is the percent of correctly classified time bins and decreasing the response time leads to increased chance of correctly classifying time bins within a cue time window. Thus, it appears that when decoding movement intentions from neural data, both accuracy and response time display a trade-off with the number of total functions, while accuracy and response time can either interact beneficially (DNN) or not significantly (SVM).

### Real-time demonstration and able-bodied comparisons

**Figure 6:**
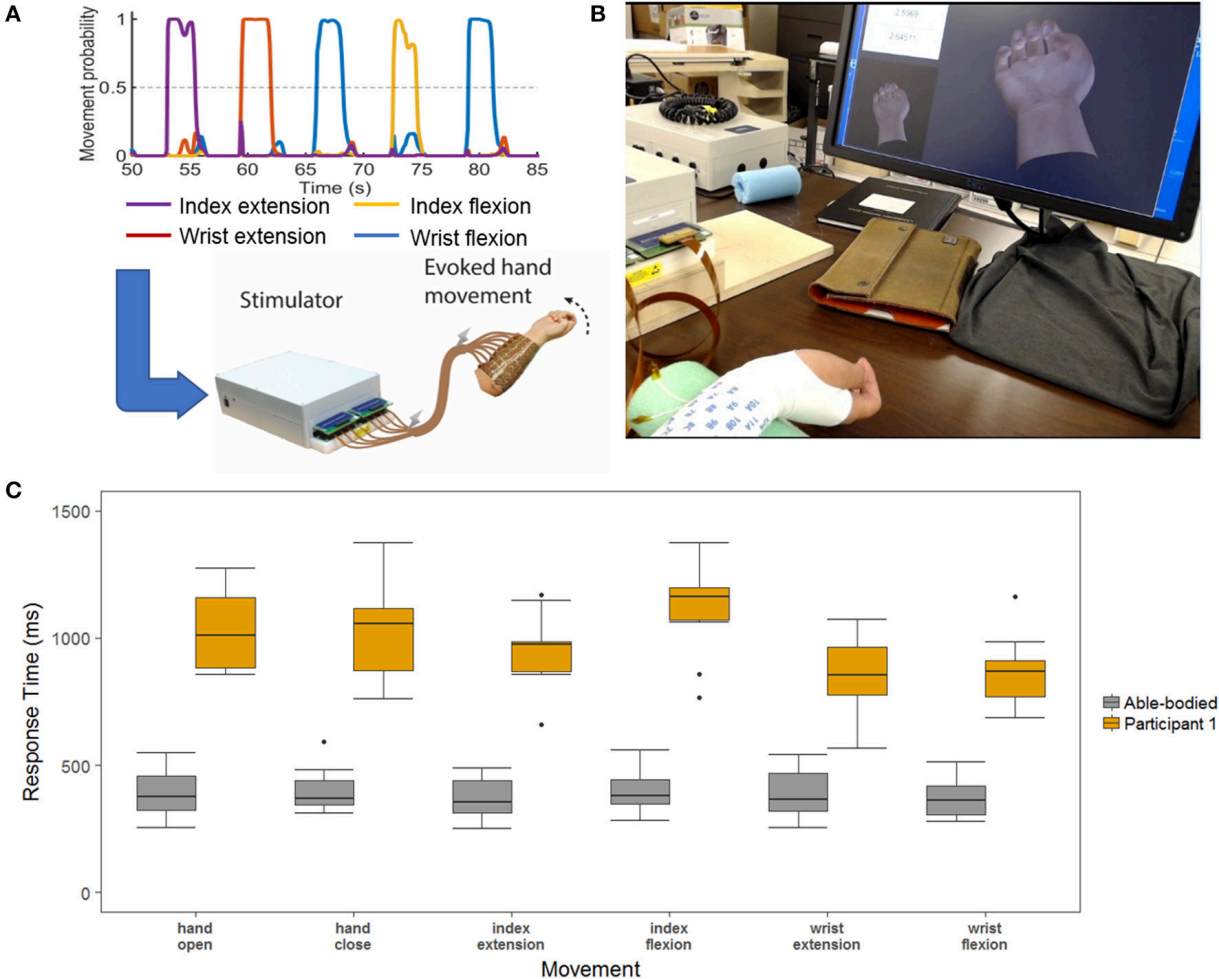
**(A)** Demonstration of data processing for the real-time demonstration. The decoder is used to decipher the neural signals to determine the desired hand movement. This information is passed to a stimulator the uses electrical signal to stimulate the participants arm to evoke the desired hand movement. **(B)** Response time layout. The two hands on the screen are the cue hand (lower-left corner) and the decoded output (center) that play in real-time. In the upper-left corner a stop watch is synched with the cue to obtain time measurements for the hand movements. **(C)** Able-bodied compared to SCI participant response times measured from videos. This is a boxplot of the response time for the six-movement task broken out by each of the hand movements. The SCI is in yellow, and the aggregate of the three able-bodied participants are in gray.

Having found that the DNN displays the highest accuracy and shortest response times as the number of movements is increased, we tested how these performance gains might translate to a real-time BCI control scenario. We designed an experiment where the study participant used the DNN decoder to control functional electrical stimulation (FES) of his paralyzed forearm. The BCI-FES system (Figure 6A) uses a flexible sleeve with up to 150 electrodes to stimulate forearm muscles and evoke movements every 100 ms based on decoder outputs as used in previous work [(Bouton et al., 2016; Sharma et al., 2016; Colachis et al., 2018) and see Methods]. When a movement is decoded, the predetermined FES pattern is triggered for that movement [as opposed to Sussillo et al. (2015) where muscle activity was directly modeled]. The participant used the BCI-FES system to perform 3 blocks of the six-movement task. Video recordings were used to determine the participants' response times for each of the cued movements (see Methods). In addition to previous sections where response time was determined by the time from cue onset to the correct movement being decoded, here we also calculated the response time from cue onset to the time when the participant visibly initiated the correct movement with his own hand. This allowed us to quantify the time to actual movement and assess how much of that delay is due to the decoder. We also recorded 3 able-bodied participants performing three blocks of the same task to quantify ideal response times. Figure 6B shows a boxplot comparing the response times of our tetraplegic participant to those of the able-bodied participants. The able-bodied participants had an aggregate mean response time of 390 ± 96 ms while our tetraplegic participant had a mean of 997 ± 224 ms. Table 1 shows the mean response times for each individual movement. Overall, these results indicate that the tetraplegic participant responds ~600 ms slower on average than our able-bodied participants.

**Table 1:** Response times for each participant by hand movement (mean ± s.d.).

**Participant**	**Index extension**	**Index flexion**	**Hand close**	**Hand open**	**Wrist flexion**	**Wrist extension**
Able-bodied (3)	365 ± 85	399 ± 86	401 ± 87	384 ± 96	373 ± 80	387 ± 96
Participant 1 Movement	936 ± 158	1,128 ± 181	1,044 ± 193	1,031 ± 154	888 ± 150	957 ± 376
Participant 1 Decoder	714 ± 107	830 ± 241	962 ± 213	1,018 ± 194	745 ± 137	600 ± 151

**Figure 7:**
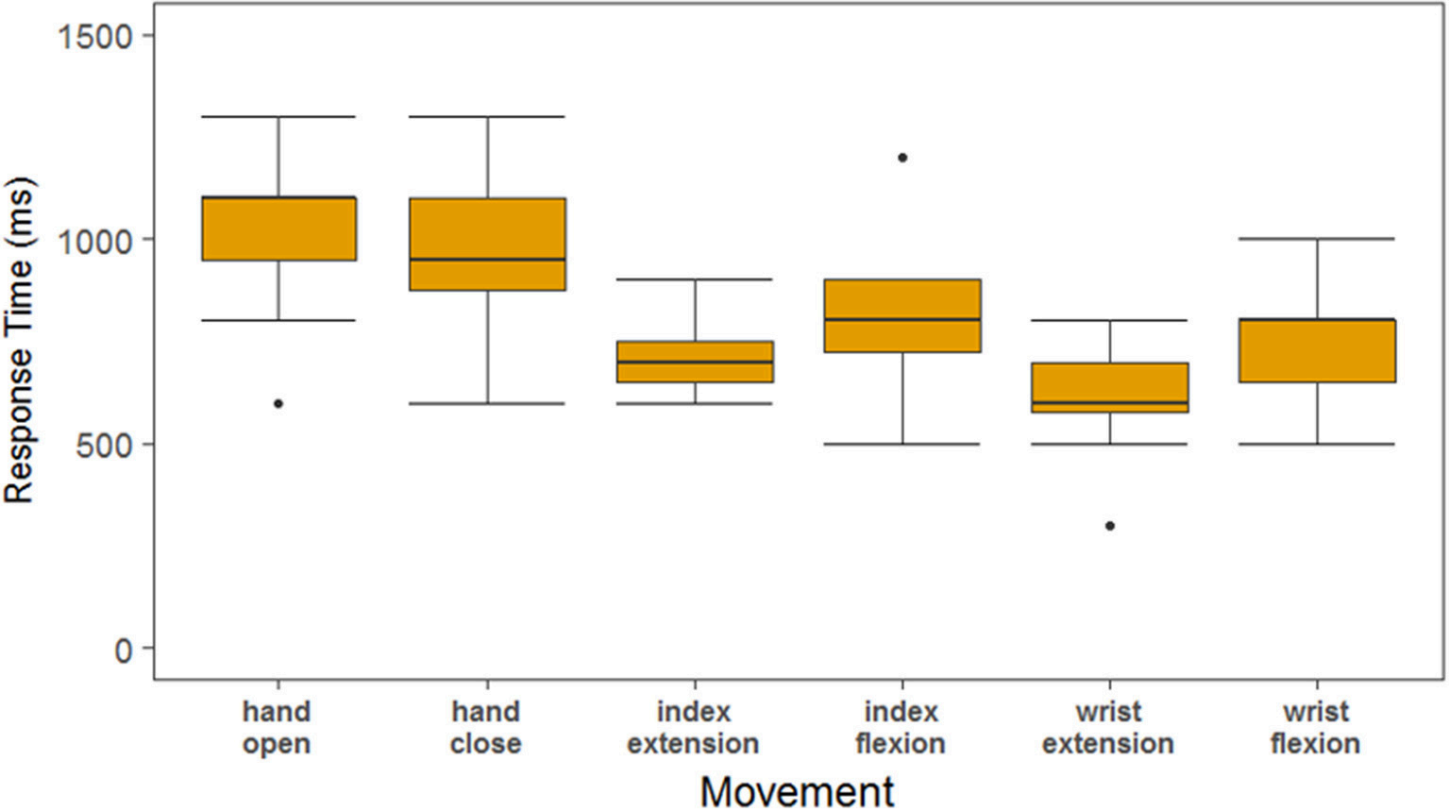
Response times of the SCI participant as measured by the decoder output. The decoder output was used to determine response times in order to separate the time that can be attributed to the algorithm vs. the rest of the system. The response times based upon the decoder are about 200 ms less than the response times determined by the video, attributing 200 ms delay to the FES system.

The tetraplegic participant's response times in this experiment are consistently higher than in the imagined movement experiments. When we computed the response times based on the decoder outputs as shown in Figure 7, the response time of the decoder was 821 ± 226 ms. In addition, there was on average a 176 ± 311 ms delay between the decoder being active and the participant's hand beginning to move that can be attributed to processing time on both the computer and stimulator, the response of the participant's muscles to the stimulation as well as any variability in the video processing. The overall success rate of the tetraplegic participant was 76.4% whereas the able-bodied participants had a success rate of 100%.

## Discussion

Recent years have seen promising advances in BCI systems aimed at reducing disability due to paralysis. Translating these advances into clinically viable systems requires thoughtful integration of patient design priorities and performance expectations with device capabilities. To that end, surveys of potential BCI users have been conducted with the goal of identifying desired system features. The results of these surveys revealed that high accuracy, fast response times, and multi-functionality are among the top prioritized system features. As the neural decoding component of the BCI system impacts all three of these features, designing and evaluating BCI decoders with these priorities in mind can facilitate the transformation of investigational systems into clinical devices and maximize the likelihood of widespread acceptance and adoption by end-users. However, designing decoders in such a manner poses a significant challenge as the user-desired priorities place competing demands on decoder performance. Characterizing the trade-offs in decoder performance that exists between the user-desired priorities is an important step toward clinically viable BCI decoders.

Here, we have explored the trade-offs that exist between accuracy, response time, and the total number of functions in decoders that infer discrete hand movement signals from intracortical neural recordings. Specifically, we trained and evaluated DNN and SVM decoders using historical data from our tetraplegic participant where he imagined performing several different hand movements. Using synthetic datasets created from this historical data, we were able to systematically investigate how increasing the number of decoded movements affects performance. Overall, we found that response time increases and accuracy decreases as the number of functions increases for both decoders, while accuracy and response time can either interact beneficially (DNN) or not significantly (SVM). Lastly, we performed an experiment where the participant used the DNN decoder in real-time to control functional electrical stimulation (FES) of the participants' paralyzed forearm, allowing him to perform six different hand/finger movements. We then compared the participants' performance with the system to that of able-bodied individuals in order to quantify the response time delay induced by the BCI-FES system.

### Data pre-processing affects decoder performance in different ways

Clearly, the manner in which the raw neural data is processed before entering the predictive model can have an impact on the performance of the model. With more sophisticated models, the impact of pre-processing may be more difficult to evaluate a priori. Our results show surprisingly divergent performance for two different decoding algorithms (SVM and DNN) based on whether the input data was initially smoothed with a 1-s boxcar filter. In our previous work, we have used the boxcar filter to smooth the neural features prior to using them in the SVM. By averaging the neural data from the current time point with the previous nine time points we reduced the variance of the input features but lessen the effect of the most recent time point which may slow down the response time. However, as the SVM only has access to the current time point and can thus make movement predictions based solely upon this single data point, including the boxcar filter effectively allows the SVM to incorporate information from prior time points, thus improving prediction accuracy. As we have shown, this smoothing is critical for the SVM–removing the boxcar filter decreases the success rate from 60.0 to 12.7%. However, the DNN does not show this same reduction in performance when the boxcar filter is removed, the success rate is not significantly different whether or not the boxcar filter is used. This can be attributed to the DNN model's ability to synthesize temporal patterns more effectively than the simpler SVM. However, as expected, using the boxcar filter does increase the response time of the DNN on average from 524 to 719 ms. Thus, data pre-processing can have a significant effect on decoder performance, but that effect can be highly dependent on the type of decoder used.

### Quantifying performance trade-offs

In the machine learning community, it is generally known that the accuracy of a classifier tends to decrease as the number of output classes increases (e.g., Fernández-Delgado et al., 2014). For BCI decoders, some work has been done to quantify the trade-offs between accuracy and number of functions (Thomas et al., 2013), as well as speed and accuracy in BCI virtual keyboard communication devices (Santhanam et al., 2006). However, a characterization of the trade-offs between accuracy, response times, and number of functions is currently lacking for BCI decoders. Using synthetic datasets, we systematically changed the number of output functions from one to six and explored how these performance criteria interact with one another for the DNN and SVM decoders. Perhaps unsurprisingly, we found that accuracy decreases as the total number of movements increases. We also found that response times tend to increase with the number of total movements. Interestingly, the performance detriment to accuracy and response times caused by increasing the number of movements was less severe for the DNN than for the SVM, with the rate of decline in accuracy being significantly less with the DNN than SVM. Additionally, we found no interaction between response time and accuracy for the SVM and a positive correlation between the two for the DNN. Though the positive correlation between response time and accuracy makes intuitive sense for BCI task presented here, it is counter to the trade-off observed for other BCI systems, such as those for virtual typing, where faster responses tend to be less accurate (Santhanam et al., 2006). This shows that the trade-offs between these three BCI performance aspects are task-specific and further work should be done to characterize the trade-offs for additional BCI tasks.

### Online performance

Surveys of potential BCI users emphasize the desire for systems to have fast response times, but did not specify target response times for BCIs that enable discrete hand movements (Collinger et al., 2013a; Huggins et al., 2015). This motivated us to perform an experiment to quantify the gap in performance between our participant and able-bodied individuals. Assuming the ideal target response time for a potential BCI user would be the response time of an able-bodied individual, the difference between BCI and able-bodied responses provides a functional metric that can be used to gauge performance. Thus, we had the participant use the DNN decoder in real time to control FES of his paralyzed forearm. We chose to use the DNN decoder as it performed better than the SVM in our offline analyses. Using the BCI-FES system, the tetraplegic participant performed the six-movement task. As the decoders all had their slowest responses when six movements were enabled, having the participant perform this task in real-time provided a “worst-case” example to compare to able-bodied responses. Our results indicate that the tetraplegic participant responds about 607 ms slower on average than our able-bodied participants. The response time can be further subdivided into the time it takes for the decoder to accurately predict the user's intent (821 ms on average) and the lag between decoder activation and forearm movement (176 ms on average). This highlights that the neural decoder response latency is only part of the total response latency. While these results are necessarily dependent on the choice of decoding algorithm and the assistive device being controlled, quantifying the difference in latency of BCI responses from that of able-bodied individuals provides a useful benchmark that is broadly applicable and can be generalized to other systems that use intracortical activity.

### Limitations

Our study is currently limited to data collected from a single participant. However, owing to the invasiveness of the surgical procedures and the experimental nature of intracortical BCIs, it is not uncommon for studies to involve only one human subject (Collinger et al., 2013b; Bouton et al., 2016; Ajiboye et al., 2017). Future work will explore how well our results generalize to other subjects. Next, the performance trade-offs we report here are specific to SVM and DNN decoders designed to infer discrete movement commands from intracortical neural data. Different tasks, decoders, or modalities (e.g., EEG) may display different trade-offs. Thus, future work should explore how these trade-offs vary across tasks, decoders, and recording modalities. Much of our analysis was performed using offline, imagined data where the participant received no feedback. Additionally, we created synthetic datasets using the offline data in order to better investigate the performance changes with increasing number of functions. Though our online demonstration provides evidence that DNN decoders may work well for online decoding with FES and visual feedback, further work is needed to verify that the trade-offs we observed carry-over to the online scenario.

## Conclusions

The accuracy, response time, and number of functions are important characteristics of a BCI system that are directly influenced by the neural decoding algorithm. In this work, we have characterized the performance trade-offs for our BCI-FES system using both SVM and DNN decoders across different combinations of pre-processing methods and number of functions. We found that the DNN has several advantages over the SVM including reduced data pre-processing requirements and less performance degradation with additional movements. Finally, we showed that a subject with tetraplegia was able to use the DNN model to control FES of his paralyzed arm to complete six different hand/wrist movements and benchmarked that performance to able-bodied individuals performing the same task. While our model training is focused on optimizing only accuracy, future work will explore whether multiple performance characteristics can be jointly optimized directly during decoder training. Optimizing a weighted average of competing objective functions has been highly successful in other fields using DNN models (e.g., Gatys et al., 2016) and may provide a more principled and tunable way to balance competing performance characteristics. As BCI technologies improve, optimizing them to simultaneously address multiple competing performance priorities will help facilitate adoption by end-users. We also suggest that such systems should aim to maximize the number of functions while simultaneously maintaining an accuracy above 90%, and a response time of <750 ms. However, we recognize that the exact metrics and targets will necessarily vary based on the type of system being used. Additionally, benchmarking against abled-bodied users for certain tasks may provide useful context for evaluating BCI systems. As more subjects are enrolled in studies for these types of assistive devices, it will become important to solicit their opinions on performance criteria, as the experience of actual users (e.g., Kilgore et al., 2001) may lead to different conclusions compared to surveys of potential users.

## Author contributions

NS, MS, GS, MB, and DF conceptualized the study and designed the experiments; NS, MS, JT, and HT performed research and data analysis; NS, MS, MB, and DF wrote the manuscript; all authors contributed to editing the manuscript.

### Conflict of interest statement

The authors declare competing interests, as they are employed by institutions that provided the funding for this work and/or have filed associated patents. NS, MS, GS, DF, JT, and HT were all employed by Battelle Memorial Institute at the time of the study and MB is employed by the Ohio State University.

## Supplementary material

Movie S1 Example video of Participant 1 performing the six-movement task with the BCI-FES system. The hand in the lower left corner of the monitor provides the cue to the participant, and the hand in the middle of the screen corresponds to the decoder predicted hand movement that is evoked with the FES.
